# Perception of Virtual Education Learning among Dental Residents and Faculty during the COVID-19 Pandemic: A Cross-Sectional Study

**DOI:** 10.3390/dj12080231

**Published:** 2024-07-23

**Authors:** Shasha Cui, Kumari Saswati Kar, Shruti Vasani, Nisreen Al Jallad, Mechelle R. Sanders, Rita Cacciato, Tong Tong Wu, Jin Xiao, Hans Malmstrom

**Affiliations:** 1 Eastman Institute for Oral Health, University of Rochester School of Medicine and Dentistry, Rochester, NY 14642, USA; shasha_cui@urmc.rochester.edu (S.C.);; 2 Department of Family Medicine, University of Rochester School of Medicine and Dentistry, Rochester, NY 14642, USA; 3 Department of Biostatistics and Computational Biology, University of Rochester School of Medicine and Dentistry, Rochester, NY 14642, USA

**Keywords:** dental education, distance education, COVID-19, dental residents and faculty, regulatory focus theory, burnout

## Abstract

Objectives: The coronavirus disease 2019 (COVID-19) pandemic prompted a rapid shift from in-person to virtual learning in dental education. This study aims to assess the perceptions of virtual education learning among dental residents and faculty and employ regulatory focus theory (RFT) to understand the impact of motivational orientations on virtual learning during the COVID-19 pandemic. Methods: In total, 46 dental residents and 10 faculty members in a dental institution participated in the study (June–August 2021). Questionnaires were used to obtain data on demographics, perceptions of virtual learning, burnout, and RFT types (promotion and prevention focus). Multiple regression analyses were used to examine factors associated with perceptions of virtual learning and burnout. Results: Overall, 70% of residents and 44% of faculty found virtual learning effective. Younger residents with less experience preferred virtual learning more than their older, experienced peers. Residents trained outside the U.S. and Canada favored in-person learning more than those trained within. Furthermore, residents with a higher promotion focus score found virtual learning more interactive for didactic courses. Additionally, 52% of residents experienced burnout, with a higher incidence among females (*p* = 0.044). Conclusions: Virtual learning is well received by dental residents and faculty, with potential for continued use post-pandemic. Future efforts should focus on creating an inclusive and supportive educational environment that meets the motivational and well-being needs of dental residents and faculty.

## 1. Introduction

The coronavirus disease 2019 (COVID-19) pandemic prompted a rapid shift from in-person to virtual learning in dental education due to the maintenance of physical distance being considered the most effective preventive strategy to limit the spread of SARS-CoV-2, combined with personal and respiratory hygiene rules in the absence of a vaccine or standardized treatment at the time.

This shift highlighted the pivotal role of e-learning and online platforms in continuing educational activities and examinations in healthcare professions education [1]. Consequently, virtual education emerged as a critical alternative to traditional in-person learning. Hence, in response to the COVID-19 pandemic, health science institutions have quickly adjusted teaching activities using e-learning and online platforms as well as examinations. Furthermore, during the COVID-19 period, the limited efficiency of traditional teaching and synchronous courses conducted didactically in online environments has brought the issue of conducting the flipped classroom method completely remotely to the fore. This situation has opened the door to the opportunity to transform the experiences gained during the COVID-19 period into post-COVID-19 gains [2]. As we reflect on the post-pandemic utilization and the lasting impact of this forced transition, a need is to reimagine the effectiveness of this current makeshift learning platform.

Some healthcare institutions continue to adopt virtual education teaching and identify effective ways to improve student engagement and interactivity [3]. For example, a qualitative study of virtual learning among 45 medical trainees from Harvard Medical School revealed high satisfaction with virtual learning during the pandemic [4]. Specifically, the majority of respondents were satisfied with virtual read-outs/virtual interdisciplinary rounds and expressed a desire to maintain key elements of virtual education in the post-pandemic period [4]. A survey among 2721 UK medical students across 39 institutions investigated the influence of online delivery modalities on facilitating medical education [5]. Survey results showed a significant increase in study time, with flexibility and interactivity being the top benefits and distractions (poor internet connection, family distractions, and the timing of the tutorials) identified as the main challenges [5]. Similarly, results from a global survey assessing participant preferences for virtual online orthodontic learning sessions concluded that the accessibility of post-session videos was instrumental in optimizing learning by improving memory retention [6]. However, engaging learners’ interests was mainly associated with the acquisition of new learning styles, knowledge, and social networking [6].

First introduced by Higgins et al. in 1997, regulatory focus theory (RFT) explained how people’s motivation towards positive outcomes (promotion focus) or away from negative outcomes (prevention focus) affects human behavior and goal achievement [7]. Participants’ responses to RFT questionnaires were used to compute scores to predict their self-regulatory orientations (promotion focus or prevention focus) [8]. Li’s study on Chinese adolescents explored the relationship between regulatory focus and learner engagement and concluded that promotion focus orientation was more predictive of learner engagement when compared with preventive focus [9]. Subjects with high promotion focus and low preventive focus exhibited higher academic self-efficacy, lower depression, greater learner engagement, and more positive adolescent development [9]. These studies suggested that RFT can offer useful insights and predictions to enhance our understanding of how students perceive motivation in dental education. However, currently, no studies have employed RFT to assess the engagement of residents in dental education, regardless of in-person or virtual modalities. This study aims to fill that gap by exploring how RFT can be applied to understand and improve dental residents’ engagement in various learning modalities, making it particularly pertinent to this research.

Furthermore, healthcare professionals already have a higher rate of burnout than the general population [10]. During the residency period, particularly, the stress level is elevated in terms of training duration and intensity, and insufficient time to adjust, leading to burnout or psychobiological exhaustion [11]. In the worst cases, burnout affects healthcare providers’ clinical judgments, the ability to communicate, and the quality of care delivery [12,13]. Amid the COVID-19 pandemic, mental health problems such as depression, anxiety, insomnia, and burnout syndrome among healthcare providers raised a heated discussion in healthcare studies [14,15,16,17].

Since limited research has been conducted to assess the perception of virtual learning among dental residents and faculty during COVID-19, and the burnout of residents, this study was designed to (a) assess the perceptions and effectiveness of virtual learning among dental residents and faculty in Upstate New York and (b) employ RFT to understand the impact of motivational orientations on virtual learning outcomes.

## 2. Materials and Methods 

### 2.1. Study Setting and Participants

From June to August 2021, 46 dental residents and 10 faculty members from an Upstate New York institute completed educational survey related to virtual educational learning. The residents were in the Advanced Education in General Dentistry (AEGD) program of the Department of General Dentistry, experienced both virtual and in-person classes. The AEGD program included five learning modalities: didactic lectures, research presentations, case presentations, literature reviews, and faculty resident meetings. The study was approved by the University of Rochester Research Subjects Review Board #STUDY00007023.

### 2.2. Questionnaire Design

The questionnaire comprised four sections: demographics, preferred online learning devices, and perceptions of virtual education in Section A; regulatory focus types in Section B; and burnout levels in Section C (Appendix A). Section A included five questions on virtual learning experiences: (1) overall feelings toward virtual learning modalities; (2) effectiveness of residents’ virtual learning experience; (3) engagement of residents during virtual learning; (4) interactions between residents and instructors in virtual learning; (5) preferred future formats. In Questions 1–4, each question is rated on a 1–5 scale, with higher scores indicating a more favorable view of virtual learning. Question 5 asked participants to choose their preferred future learning format: in-person, virtual, or hybrid.

Section B utilized a Likert scale ranging from “definitely untrue” to “definitely true” to assess regulatory focus types with Fellner et al.’s validated 10-item regulatory focus scale (RFS) questionnaire [18]. RFS is composed of two sub-scales with five questions measuring promotion focus (e.g., “I prefer to work without instructions from others”) and five questions measuring prevention focus (e.g., “Rules and regulations are helpful and necessary for me”) [19]. Scores for each scale ranged from 5 to 35, with higher scores indicating a stronger focus on the corresponding scale. A prevention or promotion score (continuous) was generated for each participant.

In Section C, participants were asked to evaluate their level of burnout, with options ranging from “having no symptoms and enjoying work” to “feeling completely burned out”. Higher scores indicated a higher level of burnout.

### 2.3. Data Analysis

A descriptive analysis was first employed to understand the perceptions of virtual learning among dental residents and faculty members. The findings were presented through a data summary, utilizing percentages and the mean ± standard deviation (SD) for a comprehensive overview. Study participants’ self-reported burnout levels were categorized into three groups based on severity. To explore factors (demographics, where they obtained dental degree, years of practice, age groups, type of program enrollment) associated with the perceptions of virtual learning, a Pearson chi-square or Fischer’s exact test was used for categorical data, and a Kruskal–Wallis test was conducted for numerical data without normal distribution. The data analysis was performed using STATA software (version 12, College Station, TX, USA).

The primary outcomes were perception of virtual learning, including overall attitude, effectiveness, engagement, interaction, and future preference. We employed a multiple logistic regression analysis to examine factors associated with these five outcomes. For these outcomes, we first combined the residents’ responses into two levels. For the overall attitude outcome, the first level was defined “Above average”, which included responses of “excellent” and “good”. The second level was defined as “Below average & average”, which included responses of “average”, “below average”, and “poor”. For effectiveness, the five-level scale was also combined into two categories: “Not effective to effective” and “More effective”. For interaction, the five-level scale was merged into two categories: “Not interactive to interactive” and “More interactive”. Similarly, for engagement and interaction, the five-level scales for these were integrated into two categories each, “Not engaged to engaged” and “More engaged” for engagement, and “Not interactive to interactive” and “More interactive” for interaction, respectively.

For the regression models of engagement and interaction, we included the following independent covariates: RFT scores (numerical) as independent variables and other factors including gender (female vs. male), the location of the DDS received (outside of the U.S./Canada vs. U.S./Canada), years of practice, and the participants’ age (20–30 yrs, 31–40 yrs, and 41–50 yrs). For the regression models of overall attitude, effectiveness, and preference of future learning modalities, we included the variables mentioned above but excluded the regulatory focus score.

In addition, we used a multiple logistic regression model to assess factors related to burnout. The covariates included were gender (female vs. male), the location of the DDS received (outside of the U.S./Canada vs. U.S./Canada), years of practice, and the participants’ age (20–30 yrs, 31–40 yrs, and 41–50 yrs). The statistical significance level was set at 5% for all analyses.

This educational improvement study used previously archived education records. Sample size calculation was not performed due to the nature of this study as a pilot study.

## 3. Results

### 3.1. Demographics

A total of 46 AEGD dental residents and 10 faculty members participated in the study. In summary, the demographics of study subjects included age group, gender, type of dental school attended, years of practice, enrolled residency programs, year of graduation, and preferred devices (Table 1). Among the residents, 41.4% were female, primarily in the 31–40 year age group (43.5%), followed by the 41–50 year age group (34.8%). In contrast, 30% of the faculty were female, with half over 60 years old and 40% of them between 41 and 50 years old. Most residents (82.6%) attended dental schools outside the U.S./Canada, while 40% of faculty attended schools in the U.S./Canada. Residents were mainly enrolled in 2-year or 3-year AEGD + MS programs. The majority of residents graduated in 2021, with the fewest (6.5%) set to graduate in 2024. For virtual learning, 97.8% of residents used desktop computers, whereas 60% of faculty preferred laptops.

### 3.2. Perceptions of Virtual Learning 

#### 3.2.1. Residents’ Overall Feelings about Virtual Learning Modalities

**Figure 1:**
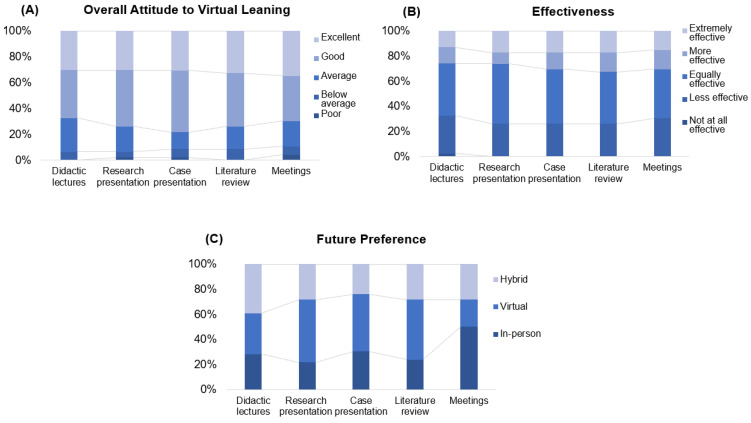
A visual analysis of perceptions of virtual learning: (**A**) a bar graph depicting the overall attitude toward different virtual learning modalities, rating them from “Excellent” to “Poor”; (**B**) a bar graph showing the perceived effectiveness of virtual learning compared with that of in-person learning, with evaluation ranging from “Extremely effective’” to “Not at all effective”; and (**C**) a bar graph representing respondents’ preferred future learning format, including hybrid, virtual, and in-person.

Overall, over 70% of participants were satisfied with virtual learning, rating their experience as above average across all five modalities (Figure 1A). Case presentations were most favored, with 78.2% receiving “Good” and “Excellent” ratings. Research presentations and literature reviews each had 73.9% above-average ratings, while didactic lectures had a balance of “Good” and “Excellent” ratings and few lower ratings. Only 4.4% rated virtual meetings as “Poor”.

Statistical analysis revealed that residents with more years of practice were less likely to favor virtual case presentations and literature reviews (Table 2). Specifically, more experienced dental practitioners showed less enthusiasm for these modalities, with odds ratios indicating decreasing preference with increasing years of practice (OR 0.80 for case presentations and OR 0.85 for literature reviews, with respective *p*-values of 0.027 and 0.047). However, factors such as gender, type of dental school attended, and age group were not statistically associated with overall feelings about virtual learning.

#### 3.2.2. Effectiveness of Residents’ Virtual Learning Experience in Comparison to That of the In-Person Learning Experience 

According to the survey, 86.5% of the participating residents indicated a positive perception of virtual learning’s effectiveness across five virtual educational modalities, with preferences varying by where they obtained the dental degree, age, and years of practice (Figure 1B). More than 73% of participants viewed research presentations, case presentations, and literature review sessions as “More effective” and “Extremely effective”. Meetings were also viewed favorably, with no participants considering them “Not at all effective”. Didactic lectures had the highest percentage of “Less effective” and “Not at all effective” ratings, indicating the relative weakness of them compared with other virtual learning components.

A significant difference was observed between the virtual method of didactic lectures and those dental residents who received their dental degree outside of the U.S. or Canada (Table 2). Residents with degrees from outside the U.S./Canada found virtual didactic lectures notably less effective [OR 0.07 (0.007, 0.65), *p* = 0.02], with a strong inclination toward in-person formats. Those aged 31–40 viewed virtual sessions as less effective [ORs 0.08 and 0.14 for case presentations and literature reviews, respectively, *p* < 0.05]. In contrast, among the residents aged 20 to 30, 50% of them preferred virtual case presentations over in-person ones. This highlights how age can impact preferences for learning modalities. Lastly, years of experience also affected attitudes toward virtual learning. Residents with more years of experience favored in-person learning over a virtual format for both case presentations (*p* < 0.05) and literature review sessions (*p* < 0.05). Gender and type of program enrollment were not statistically associated with the effectiveness of virtual learning.

#### 3.2.3. Engagement of Residents during Virtual Learning in Comparison with That during In-Person Learning

Residents reported that they were as engaged when learning through five virtual learning modalities as when courses were delivered via in-person modalities.

#### 3.2.4. Interactions between Residents and Instructors in Virtual Learning in Comparison to Those in In-Person Learning

Residents reported no difference in their interactions with instructors across five virtual learning modalities compared to in-person settings. Variables like gender, age, type of dental school, and years of practice were not statistically associated with interaction levels.

#### 3.2.5. Preferred Learning Format of Residents for Upcoming Years

Approximately 69% of residents clearly preferred incorporating virtual or hybrid elements into most learning modalities as their future learning format (Figure 1C). Among the five modalities, virtual research presentations and virtual literature review sessions were the most preferred learning methods. Notably, 50% of participants preferred in-person meetings, underscoring the importance of direct interaction in these types of meetings and classes.

A significant difference existed between the virtual method of literature review and where they received their dental degrees. The results indicated a strong preference for literature review among the participating residents [OR (95% CI) 12.04 (1.04, 139.5), *p* = 0.047] (Table 2). Even though the results did not reveal a significant difference in any of the other learning modalities, an interesting consistency emerged within foreign-trained residents. All foreign-trained participants expressed a preference for hybrid research presentations, case presentations, and literature review sessions. Variables such as gender, age, and years of practice were not statistically associated with preferred learning formats.

**Figure 2:**
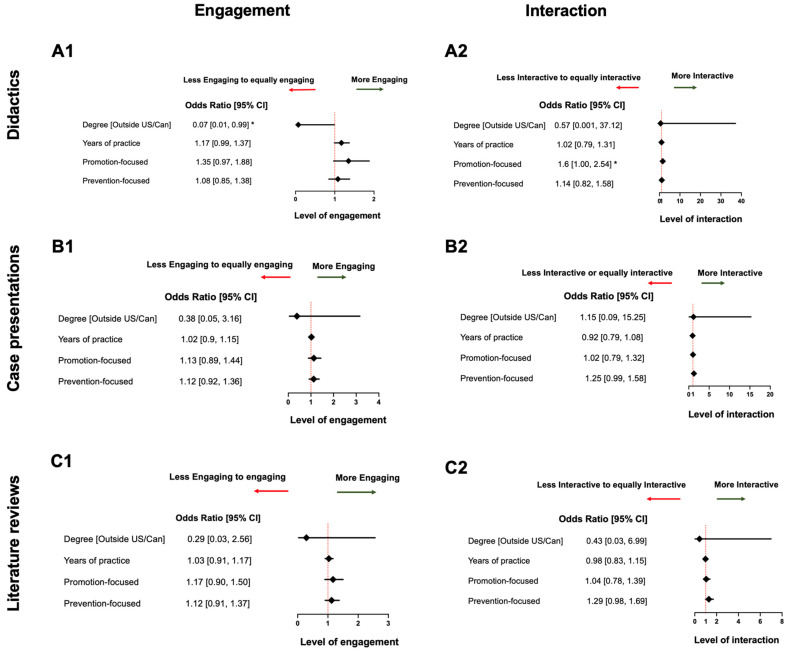
Impact of RFT, a dental degree, and years of practice on engagement and interaction forest plot of odds ratios (ORs) with 95% confidence intervals (CIs), evaluating factors affecting engagement and interaction with three different educational modalities, didactics, case presentations, and literature reviews, as perceived by participants. Each point on the plot represents the odds ratio, with the horizontal lines depicting the 95% confidence intervals. The analysis indicates that having a degree from outside the U.S./Canada is significantly associated with lower odds of engagement in didactics ((**A1**): OR 0.07, 95% CI [0.01, 0.99]). For interaction, promotion-focused activities are significantly associated with higher odds of interaction in didactics ((**A2**): OR 1.61, 95% CI [1.00, 2.54]). * Statistical significance level: 0.05. The analysis indicates that having a degree from outside the U.S./Canada, years of practice, and impact of RFT are not significantly associated with engagement and interaction in case presentations or literature reviews (**B1**–**C2**).

Figure 2 depicts a forest plot examining factors, and significant differences were mainly observed in how respondents interpreted effectiveness and interactions with online didactic dental courses. Dental residents with dental degrees from outside the U.S./Canada have a significantly lower likelihood of engaging virtual didactic lectures compared to their counterparts with U.S./Canadian dental degrees [OR (95%CI) 0.07 (0.01, 0.09, *p* < 0.05)] (Figure 2A1).

### 3.3. Regulatory Focus Theory

Each participant had a score for both prevention and promotion focus (continuous). The promotion scores ranged from a low of 15 to a high of 32, and prevention scores ranged from 19 to 35. The survey results present a view of respondents’ preferences for 10 statements in the RFS questionnaire.

**Figure 3:**
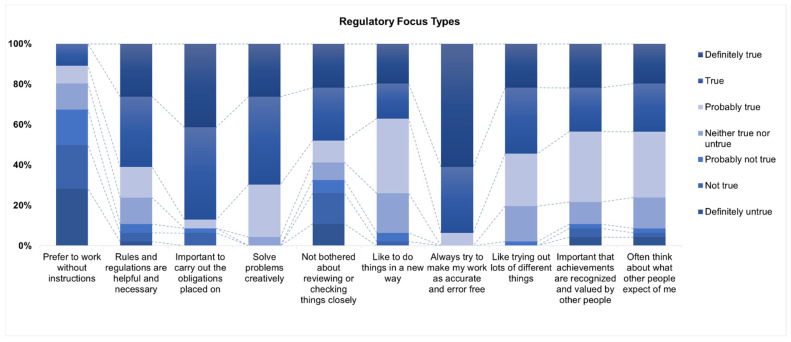
Regulatory focus types: attitudes and preferences towards work styles and a professional mindset.

A significant portion of respondents (67.4%) did not prefer to work without instructions, while only a small percentage (10.8%) agreed that rules and regulations are helpful and necessary (Figure 3). In total, 59% of them did not like being reviewed and had their work checked closely by others. More than 90% of respondents valued creativity in problem-solving, and many participants are open to innovation. All of the respondents agreed that they always try to make work accurate and error-free. The majority of participants thought social recognition and expectations from others were important to them (78.20% and 76.10%, respectively). Figure 2A2 shows that residents with a higher score for promotion RFT considered virtual didactic courses more interactive than in-person learning during COVID-19 [OR (95% CI) 1.6 (1.00, 2.54, *p* < 0.05)]. However, for other learning modalities, such as research presentations, case presentations, literature review sessions, and faculty-resident meetings, RFT scores for either promotion focus or prevention focus were not found to be significantly associated with the residents’ perceptions of virtual learning.

### 3.4. Burnout Level among the Residents

Out of the 46 respondents, 43.5% reported no burnout, 52.2% experienced occasional burnouts with some symptoms, and 4.4% felt completely burned out and frustrated at work. Gender was found to be associated with perceived burnout by residents, and the burnout rate among female residents was shown to be statistically significant [OR (95% CI) 3.8 (1.03, 13.7), *p* = 0.044] (Table 2). Where residents received a dental degree, practicing years and residents’ age were not statistically associated with burnout (*p* > 0.05). 

## 4. Discussion

### 4.1. Factors Associated with Residents’ Perceptions of Virtual Learning

Statistically significant differences in perceptions of virtual learning and effectiveness were observed in the results, and three factors were associated with these, including where dental residents obtained dental degrees outside of the U.S./Canada, the number of years of practice, and the participants’ age. 

#### 4.1.1. Residents Obtained Dental Degrees outside of the U.S./Canada

A key finding is that overall feelings about virtual education learning modalities and perceived effectiveness of virtual learning are substantially influenced by where they received their dental degrees. Foreign-trained dentists are more likely to choose traditional in-person learning formats over virtual ones than their peers. 

One of the primary reasons for their reluctance toward virtual learning is the differences in educational systems and pedagogical methods [20]. Many countries’ dental educational systems emphasize in-person lecture-based learning and hands-on clinical experience [21]. The shift to virtual learning could deviate from students’ familiar learning environment and negatively influence their levels of engagement. Another notable reason is technology familiarity [22]. Foreign-trained residents might come from countries where digital infrastructure is less developed, so they may have fewer online learning experiences in their home countries, and the transition to online learning would be more challenging for them as they need additional time to be familiar with online learning tools. Lastly, for many foreign-trained residents, creating a new professional community in a new country is as important as learning knowledge [23]. In-person interactions can provide opportunities for real and valuable engagement with peers and faculty in networking, mentorship, and intercultural communication. The finding suggests that education learning modalities should adapt to foreign-trained learners’ preferences and backgrounds, incorporating diverse teaching methods to enhance engagement. Another possible reason for the international-trained residents preferring virtual modalities is that they practiced longer before entering the program compared with the U.S. or Canada trained residents.

#### 4.1.2. Years of Practice and Age

Residents’ views on virtual education learning varied by age and years of practice. Younger, less experienced residents preferred virtual formats, while older ones with more experience favored in-person learning, particularly for literature reviews and case presentations. This finding reflects generational differences in learning styles and aligns with what the existing literature mentioned. Younger learners, born and raised in a digital age, naturally have fluency in using digital tools, which enhances their adaptability to virtual learning environments and educational innovations [24]. To accommodate this, educators should help older residents adapt by offering tech workshops, encouraging peer support, and providing materials in both digital and physical formats to create an inclusive learning environment. For example, a workshop could include sessions on how to effectively participate in virtual classrooms, how to navigate learning management systems (LMSs), and how to use digital tools for virtual collaboration and communication. The training could also cover the use of educational software, such as interactive simulations and digital textbooks, which can help older residents become more comfortable with the technology integral to modern education. Additionally, encouraging peer support through mentorship programs, where more tech-savvy co-residents can assist their peers, and providing materials in both digital and physical formats can further create an inclusive and supportive learning environment.

Our study suggests that residents found virtual education methods like case presentations and literature reviews more effective than in-person learning (*p* < 0.05). This aligns with the preference for flexible virtual curricula on platforms such as Osmosis, Bite Medicine, Becoming A Doctor, and Sustaining Medical Education in a Lockdown Environment (SMILE) of students because of their virtual and flexible curriculum [5]. However, residents still favored in-person over virtual meetings for their interactivity, supporting findings that in-person meetings are seen as more engaging than online ones [25] and are unlikely to be replaced post-pandemic [26].

### 4.2. Faculty Perceptions

Our study found no significant differences in dental faculty’s perceptions of virtual education learning, indicating that the transition to virtual platforms did not impact their teaching quality. However, future qualitative research through faculty interviews could offer deeper insights.

### 4.3. Regulatory Focus Types

Kluger and Dijk noted that medical learners often emphasize prevention, focusing on error avoidance and safety [27]. In contrast, those with a promotion focus prioritize growth and achievements [28]. Our study found no clear preference for either regulatory focus, but residents with higher promotion scores interacted more in virtual courses than in-person ones.

### 4.4. Hybrid Learning Format

Hybrid learning, which integrates online and face-to-face education, is preferred by respondents for its unique benefits. It offers students the flexibility to engage with course materials and lectures at their convenience, which is particularly beneficial for those who need to balance their studies with other responsibilities, such as work or caregiving [29]. This adaptability supports diverse learning styles and makes education more inclusive. Studies have shown that hybrid learning leads to significantly better outcomes in knowledge acquisition within health professions and effectively address time and location limitations by reaching more students without additional resource demands [30,31]. It is believed that hybrid learning enhances self-learning skills, allowing students to engage more deeply and actively in the learning process, while also improving teacher–student interactions and clarifying the material [32]. These benefits make hybrid learning a preferred approach for dental residents who appreciate the combination of flexibility, engagement, and efficient use of resources.

### 4.5. Burnout among Residents

The study found a significant correlation between gender and burnout, with female residents experiencing higher burnout rates and greater work–life dissatisfaction compared with their male counterparts. This finding aligns with previous research on gender-specific burnout issues among physicians [18,33,34]. Women are expected to be “superwomen” [35], whereas managing time between professional responsibilities and personal life can be difficult. Several factors contribute to burnout in female residents, such as a lack of supportive mentors, dual career challenges, limited childbearing years, salary inequity, fewer promotion opportunities, and higher rates of sexual harassment in the workplace [36].

To combat burnout, particularly among female dental residents, it is suggested that comprehensive wellness support, mentorship, and orientation programs be implemented [37]. Orientation programs for incoming residents should include work expectations, stress coping skills, and resources for managing stress. A formal mentorship program could focus on topics about professional development, work–life balance, well-being, and family-related challenges, rather than just work-related matters. Institutions should foster a supportive culture and enhance the well-being of women residents.

### 4.6. Limitation

The study has several limitations. The cross-sectional data from a single institution may affect generalizability, and self-reported data may introduce bias. The study has a limited sample size of 46 residents and was conducted in a single location. A bigger sample size involving multiple sites could yield a better distribution of demographics and RFT types among the study population. Further studies with a larger sample size are needed to validate the association between potential influencing factors and educational outcomes among dental residents and faculty. Additionally, we did not measure teaching quality by asking students directly. Future research should include student evaluations to provide a more comprehensive assessment of teaching effectiveness, offering valuable insights and identifying areas for improvement.

## 5. Conclusions

Virtual learning is well received by dental residents and faculty, with potential for continued use post-pandemic. Future efforts should focus on creating an inclusive and supportive educational environment that meets the motivational and well-being needs of dental residents and faculty.

## Figures and Tables

**Table 1:** Demographics–education–practice background of residents and faculty.

	Residents (*n* = 46)	Faculty (*n* = 10)
Gender (female)	41.40% (27)	30% (3)
Age group (year)		
20–30	21.7% (10)	0
31–40	43.50% (20)	10% (1)
41–50	34.80% (16)	40% (4)
51–60	0	0
>60	0	50% (5)
Dental school		
U.S./Canada	17.40% (8)	40% (4)
Outside U.S./Canada	82.60% (38)	60% (6)
Program enrolled		
1-yr AEGD	15.20% (7)	
2-yr AEGD	39.10% (18)	
3-yr AEGD + MS	37.00% (17)	
4-yr AEGD + MS	8.70% (4)	
Program graduation (year)		
2021	47.80% (22)	
2022	26.10% (12)	
2023	19.60% (9)	
2024	6.50% (3)	
Device used		
Desktop	97.80% (45)	30% (3)
Laptop	2.20% (1)	60% (6)
Tablet	0	0
Smartphone	0	10% (1)

**Table 2:** Residents’ perceptions of virtual learning and burnout level.

	Odds Ratio [95% Confidence Interval]	*p*-Value
Overall feelings about virtual learning		
*Case presentation*		
Years of Practice	0.80 [0.65,0.97]	0.027 *
*Literature review*		
Years of Practice	0.85 [0.72, 0.99]	0.047 *
Effectiveness of virtual learning		
*Didactic lectures*		
Dental degree Received Outside U.S./Canada (Yes vs. No)	0.07 [0.007, 0.65]	0.020 *
*Case presentation*		
Age group (31–40 yrs vs. 20–30 yrs)	0.08 [0.01, 0.68]	0.021 *
*Literature review*		
Age group (31–40 yrs vs. 20–30 yrs)	0.14 [0.02, 0.93]	0.041 *
Future preferred learning format		
*Literature review*		
Dental Degree Received Outside U.S./Canada (Yes vs. No)	12.04 [1.04, 139.5]	0.047 *
Overall Burnout level		
Gender (Female vs. Male)	3.8 [1.03, 13.7]	0.044 *

* Statistical significance level: 0.05. Multiple regression models were used to assess the association between the perceptions of virtual learning in terms of overall feelings, effectiveness, preferred future learning format, and burnout with independent covariates including demographics, experience in previous dental school, years of practice, and type of program enrollment. Only factors with statistical significance are presented in the table.

## Data Availability

The data used in this study are available upon reasonable request from the corresponding author.

## Appendix A

EIOH General Dentistry Virtual Learning Education Quality Improvement Questionnaire

**Please complete the following questions**

1 **Your gender**

(1) Male (2) Female (3) Non-binary

2 **Age**

(1) 20–30 (2) 31–40 (3) 41–50 (4) 51–60 (5) >60

3 **Where did you receive your DDS/DMD?**

(1) US/Canada (2) Outside of US/Canada

4 **How long have you been practicing dentistry?    Years**

5 **Which residency program are you currently in?**

(1) 1-year AEGD

(2) 2-year AEGD

(3) 3-year AEGD (Community, Urgent care, Research, Complex care, Master program)

(4) 4-year Advanced Faculty Training Program

(5) 1-year GPR

(6) 2-year GPR

(7) Preceptorship

6 **When will you graduate from your program at the EIOH?**

(1) 2021 (2) 2022 (3) 2023 (4) 2024

------------------------------------------------------------------------------

**Section A-Perception for virtual learning**

1 **What device do you use the most for virtual learning?**

(1) Laptop (2) Desktop (3) Tablet (4) Smartphone

2 **How do you feel overall about virtual learning education at EIOH AEGD?**

**Courses**	**Poor**	**Below average**	**Average**	**Good**	**Excellent**
(a) Didactic lectures					
(b) Research presentation					
(c) Case presentation					
(d) Literature review					
(e) Meetings					

3 **How effective has virtual learning been for you, comparing to in-person learning, for the following courses?**

**Courses**	**Not at all** **effective**	**Less** **effective**	**Equally** **effective**	**More** **effective**	**Extremely** **effective**
(a) Didactic lectures					
(b) Research presentation					
(c) Case presentation					
(d) Literature review					
(e) Meetings					

4 **How would you rate your engagement in virtual learning, comparing to in-person learning, for the following courses?**

**Courses**	**Not at all engaged**	**Less engaged**	**Equally engaged**	**More engaged**	**Extremely engaged**
(a) Didactic lectures					
(b) Research presentation					
(c) Case presentation					
(d) Literature review					
(e) Meetings					

5 **How would you rate your interactions with your co-residents and instructors during virtual learning, comparing to in-person learning, for the following courses?**

**Courses**	**No** **interactions**	**Less** **interactions**	**Equal** **interactions**	**More ** **interaction**	**Extremely** **more ** **interactions**
(a) Didactic lectures					
(b) Research presentation					
(c) Case presentation					
(d) Literature review					
(e) Meetings					

6 **In the coming year, what learning format would you prefer for the following courses?**

**Courses**	**In-person**	**Virtual**	**Hybrid**
(a) Didactic lectures			
(b) Research presentation			
(c) Case presentation			
(d) Literature review			
(e) Meetings			

7 **Do you have other comments for AEGD virtual learning?**

**Section B**

	**Please indicate next to each statement the extent to which each is true for you. Circle your** **response.**	**Definitely untrue**	**Not true**	**Probably not true**	**Neither true nor** **untrue**	**Probably true**	**True**	**Definitely true**
1	I prefer to work without instructions from others	0	1	2	3	4	5	6
2	Rules and regulations are helpful and necessary for me	0	1	2	3	4	5	6
3	For me, it is very important to carry out the obligations placed on me	0	1	2	3	4	5	6
4	I generally solve problems creatively	0	1	2	3	4	5	6
5	I'm not bothered about reviewing or checking things really closely	0	1	2	3	4	5	6
6	I like to do things in a new way	0	1	2	3	4	5	6
7	I always try to make my work as accurate and error free as possible	0	1	2	3	4	5	6
8	I like trying out lots of different things, and am often successful in doing so	0	1	2	3	4	5	6
9	It is important to me that my achievements are recognized and valued by other people	0	1	2	3	4	5	6
10	I often think about what other people expect of me	0	1	2	3	4	5	6

**Section C**

**Overall, based on your definition of burnout, how would you rate your level of burnout?**

A I enjoy work I have no symptoms of burnout.
B Occasionally I am under stress and I don’t have as much energy as I once did, but I don’t feel burned out.
C I am definitely burned out and have one or more symptoms of burnout, such as physical and emotional exhaustion.
D The symptoms of burnout that I’m experiencing won’t go away. I think about frustration at work a lot.
E I feel completely burned out and often wonder if I can go on. I am at the point where I made some changes or may need to seek some sort of help.
